# Supplementary material for: Comparison of greenhouse gas emissions associated with the construction of timber, concrete, and steel check dams in Akita, Japan: An input-output analysis
Source: PLoS One. 2025 Jan 15;20(1):e0316153. doi: 10.1371/journal.pone.0316153 (PMC11734949; doi:10.1371/journal.pone.0316153)
Supplement: S1 Table — (PDF) [file pone.0316153.s001.pdf]

Materials	Industrial sectors
Processed square timber (Japanese cedar)	Timber
Polyethylene pipe	Plastic products
Deformed steel bar	Hot rolled steel
Anti-absorption mat	Textile products
Ggasoline	Petroleum refinery products
Light oil	Petroleum refinery products
Sandbag	Textile products
Turf	Crop cultivation
Timber signboard	Timber
Blended oil	Petroleum refinery products
Dam nameplate (timber)	Timber
Chain lubricant	Petroleum refinery products
Lag screw	Bolts, nuts, rivets and springs
Metal washer	Bolts, nuts, rivets and springs
Four-sided chamfered timber (Japanese cedar)	Timber
Threaded rod	Bolts, nuts, rivets and springs
Hex bolt	Bolts, nuts, rivets and springs
Coupling nut	Bolts, nuts, rivets and springs
Nut	Bolts, nuts, rivets and springs
Square washer	Bolts, nuts, rivets and springs
Gabion Stone	Miscellaneous mining industry
Vegetation mat	Crop cultivation
