# Supplementary material for: Comparison of greenhouse gas emissions associated with the construction of timber, concrete, and steel check dams in Akita, Japan: An input-output analysis
Source: PLoS One. 2025 Jan 15;20(1):e0316153. doi: 10.1371/journal.pone.0316153 (PMC11734949; doi:10.1371/journal.pone.0316153)
Supplement: S2 Table — (PDF) [file pone.0316153.s002.pdf]

Materials	Industrial sectors
Polyethylene pipe	Plastic products
Polyvinyl chloride pipe	Plastic products
Pipe filter	Plastic products
Ggasoline	Petroleum refinery products
Light oil	Petroleum refinery products
Sandbag	Textile products
Turf	Crop cultivation
Timber signboard	Timber
Blended oil	Petroleum refinery products
Dam nameplate (aluminum)	Non-ferrous metal products
Chain lubricant	Petroleum refinery products
Timber panel	Timber
Ready-mixed concrete	Ready mixed concrete
Regeneration crusher run	Miscellaneous ceramic, stone and clay products
Vegetation mat	Crop cultivation
