# Supplementary material for: Comparison of greenhouse gas emissions associated with the construction of timber, concrete, and steel check dams in Akita, Japan: An input-output analysis
Source: PLoS One. 2025 Jan 15;20(1):e0316153. doi: 10.1371/journal.pone.0316153 (PMC11734949; doi:10.1371/journal.pone.0316153)
Supplement: S3 Table — (PDF) [file pone.0316153.s003.pdf]

Materials	Industrial sectors
Polyethylene pipe	Plastic products
Anti-absorption mat	Textile products
Ggasoline	Petroleum refinery products
Light oil	Petroleum refinery products
Sandbag	Textile products
Turf	Crop cultivation
Timber signboard	Timber
Gabion	Hot rolled steel
Steel flexible frame	Coated steel
Blended oil	Petroleum refinery products
Dam nameplate (aluminum)	Non-ferrous metal products
Chain lubricant	Petroleum refinery products
Gabion Stone	Miscellaneous mining industry
Vegetation mat	Crop cultivation
