# Supplementary material for: Comparison of greenhouse gas emissions associated with the construction of timber, concrete, and steel check dams in Akita, Japan: An input-output analysis
Source: PLoS One. 2025 Jan 15;20(1):e0316153. doi: 10.1371/journal.pone.0316153 (PMC11734949; doi:10.1371/journal.pone.0316153)
Supplement: S4 Table — (PDF) [file pone.0316153.s004.pdf]

| Effects | Sector | Greenhouse gas emissions |
|---|---|---|
| **Direct effects** | Road transport (except self-transport) | 2,245 |
| | Timber | 908 |
| | Bolts, nuts, rivets, and springs | 419 |
| | Crop cultivation | 361 |
| | Petroleum refinery products | 139 |
| | Others | 508 |
| **Indirect effects** | Electricity | 6,136 |
| | Pig iron and crude steel | 5,758 |
| | Self-transport | 2,044 |
| | Logs | 1,873 |
| | Road transport (except self-transport) | 744 |
| | Others | 2,994 |
| **Total** | Electricity | 6,136 |
| | Pig iron and crude steel | 5,758 |
| | Road transport (except self-transport) | 2,988 |
| | Self-transport | 2,044 |

| | |
|---|---|
| Logs | 1,873 |
| Others | 5,329 |
