# Supplementary material for: Comparison of greenhouse gas emissions associated with the construction of timber, concrete, and steel check dams in Akita, Japan: An input-output analysis
Source: PLoS One. 2025 Jan 15;20(1):e0316153. doi: 10.1371/journal.pone.0316153 (PMC11734949; doi:10.1371/journal.pone.0316153)
Supplement: S5 Table — (PDF) [file pone.0316153.s005.pdf]

**Effects**	**Sector**	**Greenhouse gas emissions**
**Direct effects**	Ready-mixed concrete	1,041
	Road transport (except self-transport)	582
	Crop cultivation	144
	Plastic products	103
	Timber	91
	Others	158
**Indirect effects**	Cement	38,372
	Electricity	5,341
	Self-transport	1,246
	Road transport (except self-transport)	474
	Petroleum refinery products	245
	Others	1,224
**Total**	Cement	38,372
	Electricity	5,341
	Self-transport	1,246
	Road transport (except self-transport)	1,056

Ready-mixed concrete	1,041
Others	1,965
