# Supplementary material for: Comparison of greenhouse gas emissions associated with the construction of timber, concrete, and steel check dams in Akita, Japan: An input-output analysis
Source: PLoS One. 2025 Jan 15;20(1):e0316153. doi: 10.1371/journal.pone.0316153 (PMC11734949; doi:10.1371/journal.pone.0316153)
Supplement: S6 Table — (PDF) [file pone.0316153.s006.pdf]

Effects	Sector	Greenhouse gas emissions
**Direct effects**	Coated steel	1,998
	Road transport (except self-transport)	507
	Hot-rolled steel	376
	Miscellaneous mining industry	317
	Petroleum refinery products	170
	Others	313
**Indirect effects**	Pig iron and crude steel	16,824
	Electricity	8,978
	Cold-finished steel	1,363
	Hot-rolled steel	1,209
	Coal products	1,136
	Others	2,610
**Total**	Pig iron and crude steel	16,824
	Electricity	8,978
	Coated steel	2,000
	Hot-rolled steel	1,585

Cold-finished steel	1,363
Others	5,050
