# Supplementary material for: Comparison of greenhouse gas emissions associated with the construction of timber, concrete, and steel check dams in Akita, Japan: An input-output analysis
Source: PLoS One. 2025 Jan 15;20(1):e0316153. doi: 10.1371/journal.pone.0316153 (PMC11734949; doi:10.1371/journal.pone.0316153)
Supplement: S8 Table — (PDF) [file pone.0316153.s008.pdf]

Sector	Net reduction ($kg\text{-}CO_2eq$)	Sector	Net emissions ($kg\text{-}CO_2eq$)
Pig iron and crude steel	−11,066	Road transport (except self-transport)	2,091
Electricity	−2,842	Logs	1,871
Coated steel	−1,959	Self-transport	1,118
Cold-finished steel	−1,146	Timber	897
Hot-rolled steel	−1,062	Bolts, nuts, rivets, and springs	421
Coal products	−737	Crop cultivation	296
Others	−399	Others	846
Total	−19,213	Total	7,540
