# Supplementary material for: Comparison of greenhouse gas emissions associated with the construction of timber, concrete, and steel check dams in Akita, Japan: An input-output analysis
Source: PLoS One. 2025 Jan 15;20(1):e0316153. doi: 10.1371/journal.pone.0316153 (PMC11734949; doi:10.1371/journal.pone.0316153)
Supplement: S9 Table — (PDF) [file pone.0316153.s009.pdf]

Sector	Net reduction ($kg\text{-}CO_2eq$)	Sector	Net emissions ($kg\text{-}CO_2eq$)
Cement	−4,499	Pig iron and crude steel	5,695
Ready-mixed concrete	−1,202	Road transport (except self-transport)	1,932
Miscellaneous ceramic, stone, and clay products	−65	Logs	1,685
-	-	Timber	808
-	-	Self-transport	798
-	-	Electricity	795
Others	0	Others	2,828
Total	−4,980	Total	14,540
